# The Influence of 3D Printing Parameters on Adhesion between Polylactic Acid (PLA) and Thermoplastic Polyurethane (TPU)

**DOI:** 10.3390/ma14216464

**Published:** 2021-10-28

**Authors:** Emila Brancewicz-Steinmetz, Jacek Sawicki, Paulina Byczkowska

**Affiliations:** Institute of Materials Science and Engineering, Lodz University of Technology, Stefanowskiego 1/15, 90-924 Lodz, Poland; 211599@edu.p.lodz.pl (E.B.-S.); paulina.byczkowska@p.lodz.pl (P.B.)

**Keywords:** FDM, multi-material printing, roughness, shear strength, TPU, PLA

## Abstract

A 3D printer in FDM technology allows printing with two nozzles, which creates an opportunity to produce multi-material elements. Printing from two materials requires special consideration of the interface zone generated between their geometrical boundaries. This article aims to present the possibility of printing with PLA and TPU using commercially available filaments and software to obtain the best possible bond strength between two different polymers with respect to printing parameters, surface pattern (due to the material contact surface’s roughness), and the order of layer application. The interaction at the interface of two surfaces of two different filaments (PLA-TPU and TPU-PLA) and six combinations of patterns were tested by printing seven replicas for each. A total of 12 combinations were obtained. By analyzing pairs of samples (the same patterns, different order of materials), the results for the TPU/PLA samples were better or very close to the results for PLA/TPU. The best variants of pattern combinations were distinguished. Well-chosen printing parameters can prevent a drop in parts efficiency compared to component materials (depending on the materials combination).

## 1. Introduction

Rapid prototyping is a promising method that can support the efficient production of models and prototypes [1,2,3]. Additive manufacturing (AM) technologies allow components with adjustable geometry and mechanical properties [4,5,6] that can be used in a wide variety of industry sectors to be obtained [7]. Fused deposition modelling (FDM), also known as fused filament fabrication (FFF), is an AM process based on extrusion, where material as a filament is melted and selectively dispensed through a fine nozzle layer upon layer according to the slice model [8,9,10,11,12,13,14]. 

FDM technology assumes the use of thermoplastic materials for printing. Polylactic acid (PLA) material is a biodegradable material with a low shrinkage rate and good stability when printing large-size models [9,15,16,17]. Thermoplastic polyurethane (TPU) is one of the most versatile engineering thermoplastics materials with elastomeric properties [18,19,20,21]. TPU is a material in which linear polymer chains accomplish straight lines with flexible and rigid chains connected through covalent bonding [22]. TPU is an elastic elastomer with high performance and current consumption level and can provide a range of combinations of physical properties that can be adapted to many uses [23].

Combinations of TPU with PLA have been investigated in the relevant literature. Thermoplastic polyurethane and polylactic acid pellets were blended by hot lamination, which helped obtain distinct morphological structures and an analogically better shape-memory effect [24]. Producing blends via the solvent casting technique demonstrates the feasibility of producing a biocompatible scaffold based on TPU and PLA blends. This method has the potential to be applied in tissue engineering [25]. Considering the FDM 3D printing method, there is an option to prepare a custom filament consisting of TPU and PLA; higher TPU content results in a more outstanding tensile toughness. When using a ready-made filament for printing, the appropriate printing parameters for the material should be selected [16], and with the use of one printing nozzle, standard printing is performed.

Hu et al. [26] investigated polymer mixtures applied to form hernia meshes. This indicates a growing interest in the possibilities of combining polymers in additive production. PLA, thanks to its biocompatibility, is used in biomedicine. Liu et al. [27] modified PLA by adding TPU; this modification increased the elasticity of the electrospun fiber. Research has demonstrated its favorable morphology, hydrophilicity, and high biocompatibility. In 3D printing, Yoojung et al. [28] modeled the shape and composition of the yarn and produced it from PLA and TPU fibers, showing a better elasticity of the TPU fibers. Thus, there is the potential for a wide application of these methods in the clothing industry.

A 3D printer in FDM technology allows printing with two nozzles, which creates an opportunity to produce multi-material elements [1,2]. Printing from two materials requires special consideration of the interface zone generated between their geometrical boundaries. Lopes et al. have proven the negative influence of a geometrical boundary interface between the same material printed from two extrusion heads. The lack of chemical affinity between materials worsens the effect, and a more prominent decrease in Young’s modulus and tensile strength is obtained [29]. Printing from more than one material allows multicolored elements to be created and allows elastic mechanical properties to be obtained, which could be helpful in areas such as anatomical modeling and soft robotics [30,31,32,33,34]. As Tamburrino et al. proved, the interlayer adhesion strength is influenced by the order of printed materials, infill, process temperature, and slicing parameters [35]. According to FDM printing, as a layered production method, the surface finish of the building part is inevitably relatively rough, and this problem is raised in the broad application of the FDM process in different industries [36]. Solutions to improve the surface finish in FDM 3D printing were investigated and proposed. Anitha et al. showed that layer thickness has a greater influence on surface roughness than speed deposition and road trip parameters. Garg et al. studied the vapor treatment process on parts printed in different orientations, all of which helped improve the components’ surface finish [37]. The method of the elaborate prediction of the surface roughness expression was presented by Ahn et al. [38].

According to increasingly stringent environmental regulations imposed on the automotive sector and the expected depletion of petrochemical resources, eco-friendly alternative solutions are being sought. The interest to use next-generation bioplastics and biocomposites as novel vehicle components has increased continuously. In order to compete with petroleum-sourced plastics, the properties of PLA must be improved. Durable applications of PLA have been significantly limited by its inherent brittleness [39,40]. The possibility of combining two materials in one process influences the additive production and the achievement of better mechanical properties of prints. The challenge, however, is a satisfactory interlayer connection, which has been analyzed in this article. 3D printing provides a wide range of products in personal and health care (for example, elements with cut-resistant properties) [41]. A combination of polymers can be used to create protective barriers on clothing. Thus, providing protection and slip resistance [42,43].

Additive manufacturing with multiple materials can contribute to a, more straightforward, faster, and more efficient production process for components used in many industries. Multi-material printing in a one-step production process can be used wherever mechanical strength, resistance to damage, vibration damping, and thus plasticity are required. The hardness of the PLA phase can ensure load absorption, and the softer TPU phase can limit cracks.

Producing with additive methods allows specific models to be created and, depending on expectations, it is possible to predict and modify the element surface. This article aims to present the possibility of printing with PLA and TPU using commercially available filaments and software to obtain the best possible bond strength between two different polymers with respect to printing parameters and surface patterns (due to the roughness of the material contact surface and the order of layer application). 

## 2. Materials and Methods

### 2.1. Materials

For the test, the Ultimaker PLA red- RAL 3020 and Fillamentum Flexfill TPU 98A Natural filaments with a diameter of 2.85 mm were used. Referring to the technical datasheet, the mechanical properties of the filaments are presented in Table 1.

### 2.2. Samples

**Figure 1:**
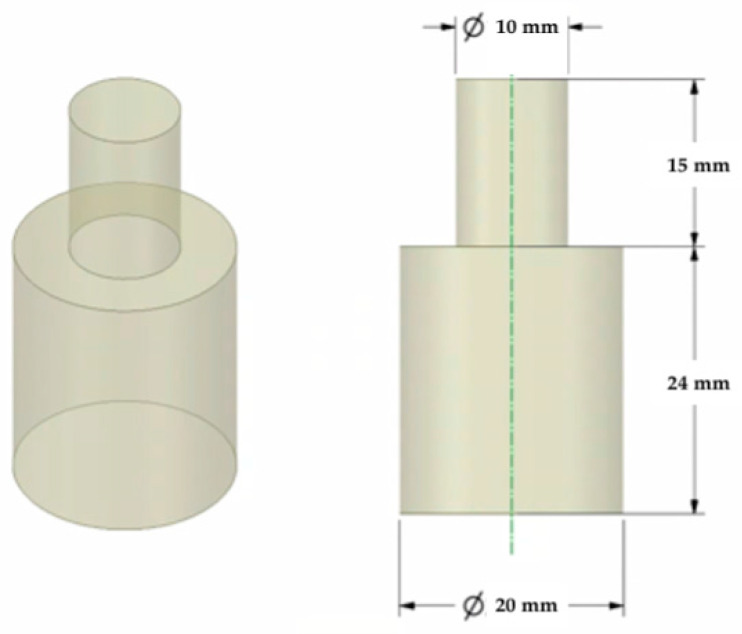
The 3D model of the sample used for the shear test.

A 3D model was created using Ansys Workbench software with the SpaceClaim module (Ansys Inc., Canonsburg, PA, USA). The model shown in Figure 1 consisted of two solid figures (coaxial cylinders used to represent the shear strength test).

**Figure 2:**
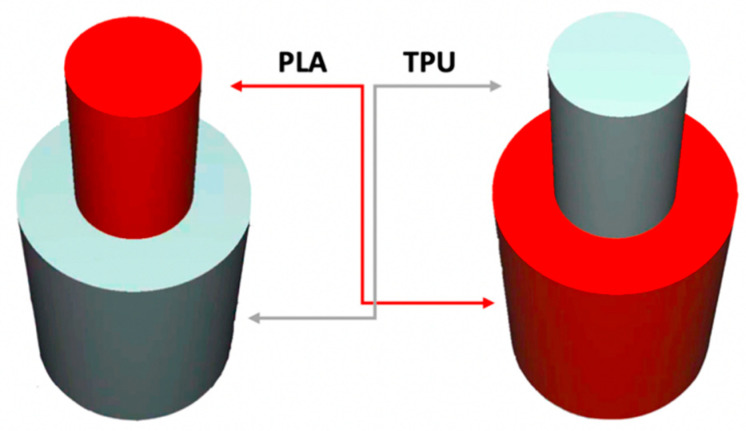
The material printing order for each pattern configuration.

To investigate the most durable connection of two materials using the 3D printing method, the surface layer pattern was changed. Modifications of external layers were made using the Cura software. The file, with each one of two parts of the sample, was uploaded to Cura software. The printing parameters were set for each part and, subsequently, two parts were merged to form one sample. Two different configurations were used to check the influence of the adhesion strength against the material printing order (Figure 2). The model of each sample, with the specified surface layer conditions, was printed in two versions. One version comprised of a series, and seven identical samples were printed in one series.

### 2.3. Printing Parameters

Experimental tests were run using the Ultimaker 3D printer with two nozzles and the Ultimaker Cura software for generating the specified print parameters. The nozzles used in both extrusion heads had a die diameter of 0.4 mm. PLA filament was placed in the first position in the printer, and TPU on the second. The printing parameters were constant for all samples (Table 2), excluding the surface layer, where the top and bottom patterns were changed. 

**Figure 3:**
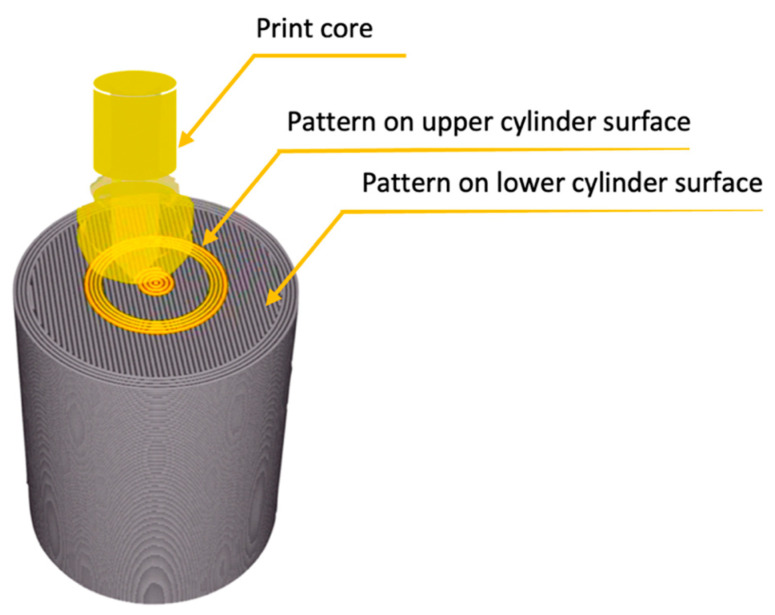
The path of filament application through the Print core, the scheme shows the application of the first layer of the upper cylinder; image obtained in Cura software (Layer View).

Layers involved in the interface adhesion are the top and the bottom layers. The Ultimaker Cura software allowed three different top and bottom patterns: *Lines*, *Concentric,* and *ZigZag*. This study compared two patterns: *Lines* and *Concentric* in different configurations. An example of the configuration and the method of applying the second material layer through the printer nozzle is shown in Figure 3.

**Figure 4:**
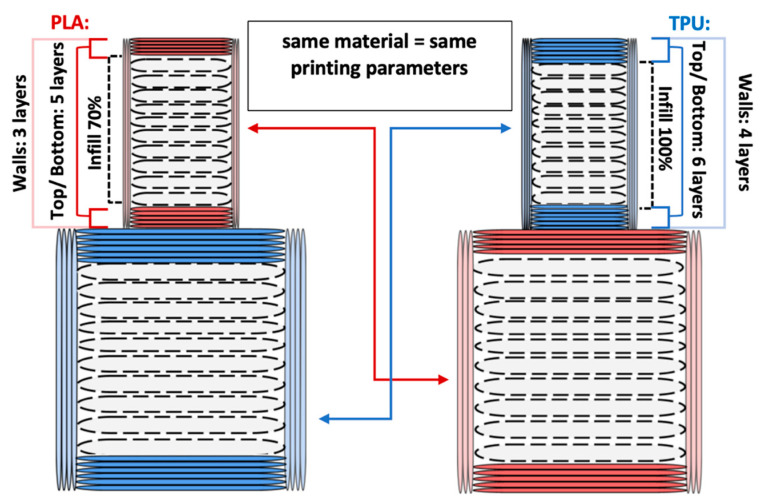
Graphical representation of sample configurations. Each part is divided into three zones: walls, top/bottom layers, and intermediate infill for both materials: thermoplastic polyurethane (blue) and polylactic acid (red).

The printing parameters specify the number of external layers, walls, and infill for TPU and PLA. The number of external layers was fixed at five for PLA and six for TPU in order to focus on the influence of the pattern used in each layer. Walls were printed in three lines for PLA and four lines for TPU. Intermediate layers were printed using 70% infill density for PLA and 100% density for TPU. However, the walls and the top/ bottom layers were always printed with 100% infill. A graphical representation of the wall and layer configurations of the sample can be seen in Figure 4.

External layers are elements on the top and bottom of the sample planes, forming a series of layers that surround the infill. The walls are a printed outer part that surrounds the circumference of the cylinders on each layer. The number of outer layers is different for TPU and PLA. One additional layer of TPU has been added to achieve a better mapping and development of the surface pattern. This was carried out due to the material’s properties and its plasticity. The assumption was to achieve an even surface representing the applied surface pattern.

Samples were printed in six pattern configurations, each in two material versions (Figure 2 and Figure 4). A list of 3D models of samples with force point variants, pattern configurations, and the number of printed samples are presented in Table 3.

### 2.4. Adhesion

**Figure 5:**
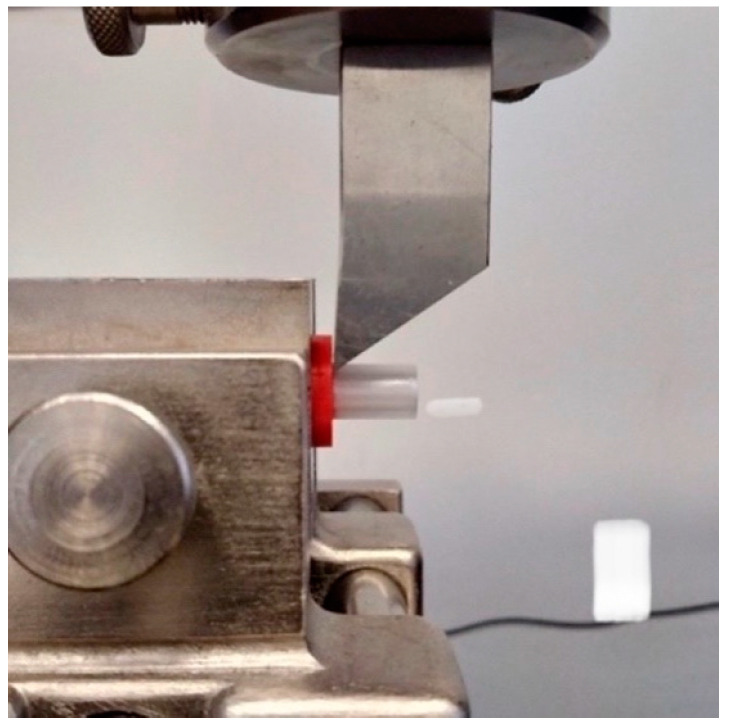
The setup for adhesion tests was performed using a shear test machine with a placed sample made of PLA as a bottom and TPU as a top cylinder.

The shear test (Figure 5) was carried out on a universal testing machine (from Zwick/Roell), according to the rules for rigid cellular plastics (ISO 1922:2018). The machine knife displacement rate was set to 1 mm/min (within the recommended range). An adhesion test was performed as described by J. Taczała et al. when investigating the bond strength between two polymers [46]. Moreover, each surface pattern configuration was analyzed in terms of its pattern directly on the cylinder surface about the force load affecting it. When there was a *Lines* pattern on the surface of the lower cylinder, the force load was applied perpendicular to this pattern. When there was a *Concentric* pattern on the lower cylinder and *Lines* on the upper, the force was applied perpendicular to the pattern on the surface on the upper cylinder. The force was applied without reference to the direction when a Concentric pattern appeared on both cylinders and, thus, the contact surfaces. 

### 2.5. Roughness

**Figure 6:**
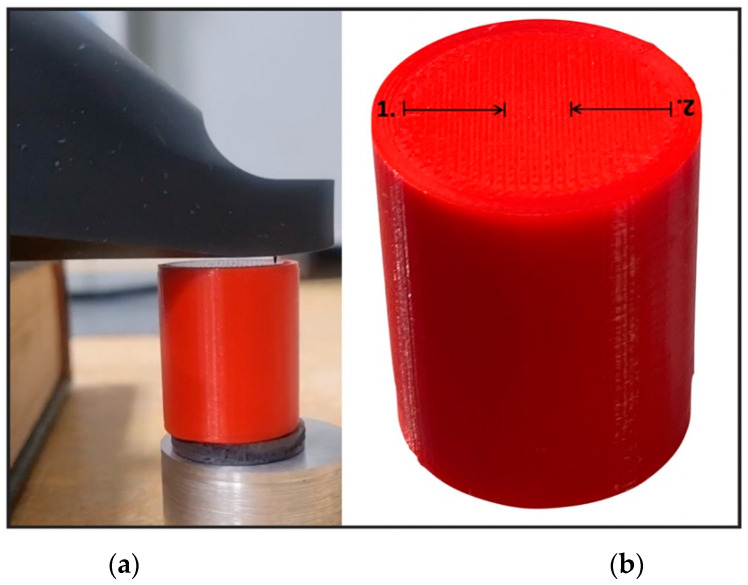
(**a**) The setup for the roughness test; (**b**) mapping distance during the roughness measurement.

To measure the roughness of the samples, lower sample cylinders were additionally printed for each pattern. Four independent samples were created. TPU cylinders with Lines and Concentric pattern and PLA cylinders with Lines and Concentric pattern. Roughness was measured using Hommel-Etamic T8000 and according to PN-EN ISO 4287:1999. The measuring setup is shown in Figure 6a. The measurements were made at a mapping distance of 4 mm from the edge of the sample (excluding outer walls) to the center, perpendicular to the pattern lines (Figure 6b).

Surface roughness parameters (Sa and Sq) were measured by a 3D optical profilometr (Leica Microsystems DCM8). Images were taken over a 2 × 1.8 mm area of the side surface. Surface roughness was analysed by the 3D Optical Surface Me-trology System Leica DCM 3D according to ISO 25178:2019.

## 3. Results

The samples were tested in two ways: the roughness of the outer surfaces on the lower cylinders were investigated and the adhesion between polymers surfaces was tested. Data obtained by measurements were tested using a single factor ANOVA test statistical significance assumed at a level of *p* < 0.05. Statistical analysis was the determination of mean and median and standard deviation (SD).

### 3.1. Adhesion

The interaction at the interface of two surfaces of two different filaments (PLA-TPU and TPU-PLA) and six combinations of patterns were tested by printing seven replicas for each; a total of twelve combinations were obtained. Eighty-four samples were assumed to be printed. Unfortunately, an attempt to print samples (N°−3) that had an angular line pattern displacement–as a bottom (PLA) interaction surface *Lines* pattern with an angle of 0° and as a top (TPU) interaction surface *Lines* pattern with an angle of 90°–failed. Four attempts were made to print these samples, but each was a failure. The failure was presumably related to insufficient adhesion of the materials with the patterns on the contact surfaces. The lower parts of the sample were printed unreservedly. Still, the problem appeared when applying the first TPU layer; the material was detaching from the samples and dragging on the nozzle. No deviations from the assumption were observed when printing the remaining batches of samples.

**Figure 7:**
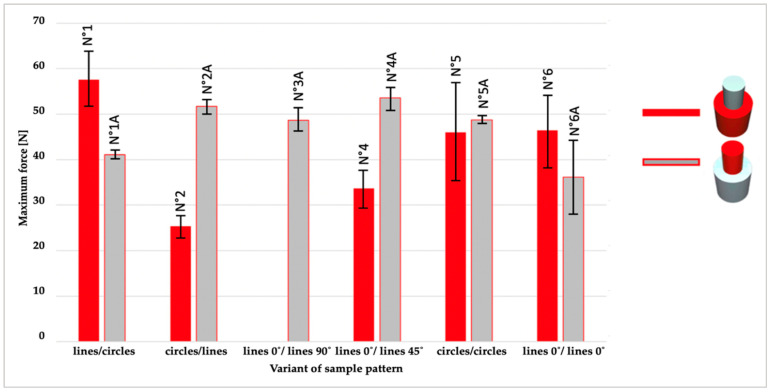
An overview of maximum force for 11 series of samples (different configuration of patterns and material sequence).

**Figure 8:**
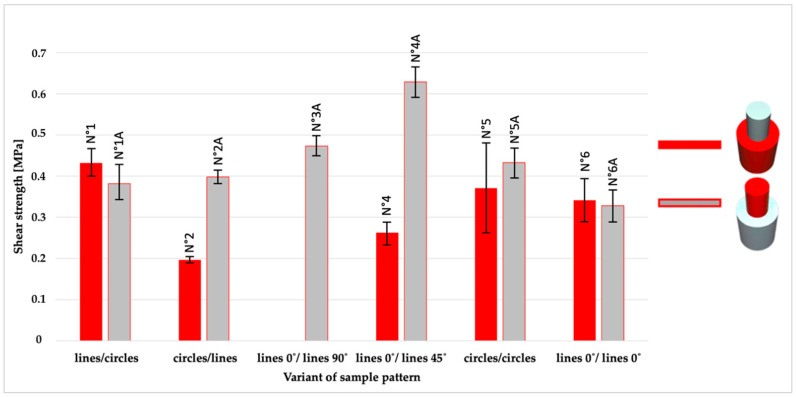
An overview of average shear strength for 11 series of samples (different configuration of patterns and material sequence).

**Figure 9:**
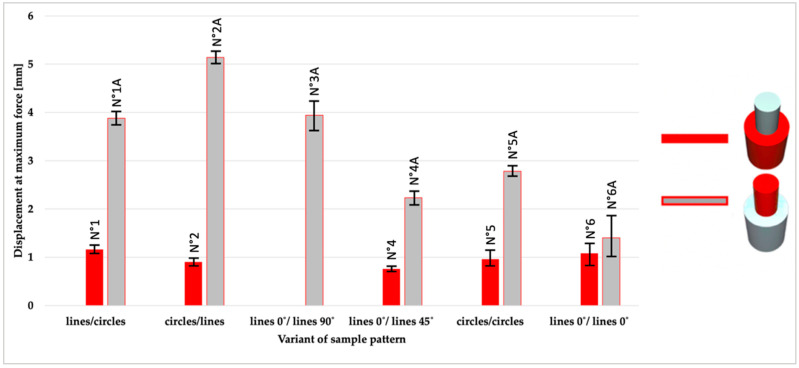
An overview of average displacement for 11 series of samples (different configuration of patterns and material sequence).

A compilation of the arithmetic means obtained from five measurements (the results of the extremes were rejected) for each series of samples are shown in Figure 7 (maximum force), while Figure 8 shows the shear strength and Figure 9 the displacement.

### 3.2. Roughness

**Figure 10:**
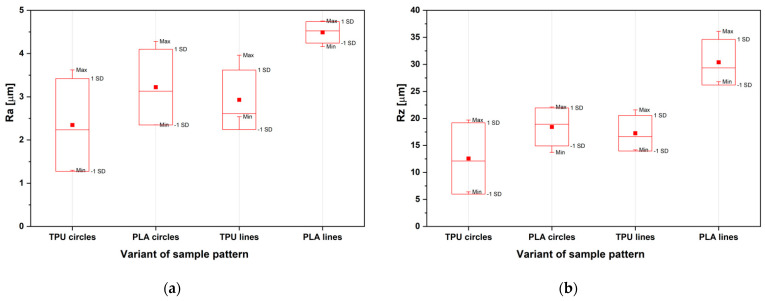
Roughness results measured by Hommel-Etamic T8000, according to PN-EN ISO 4287:1999 (**a**) Ra parameter in micrometers (**b**) Rz parameter in micrometers.

Two profile roughness parameters were analyzed: Ra, as an average length between the peaks and valleys and the deviation from the mean line on the surface within the sampling length; and Rz, as a vertical distance from the highest peak to the lowest valley within five sampling lengths and averages the distance is averaged [47]. Figure 10 shows the results: Ra (Figure 10a) and Rz (Figure 10b) roughness for the different samples, TPU and PLA, with both lines and circles on outer surfaces.

**Figure 11:**
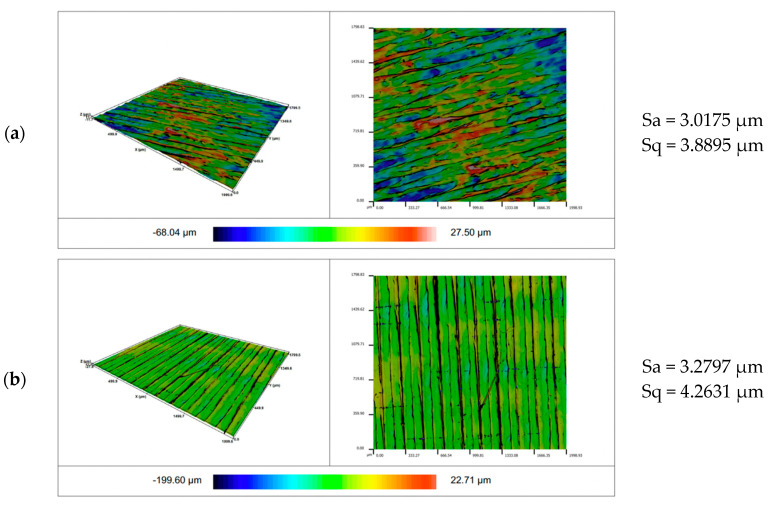

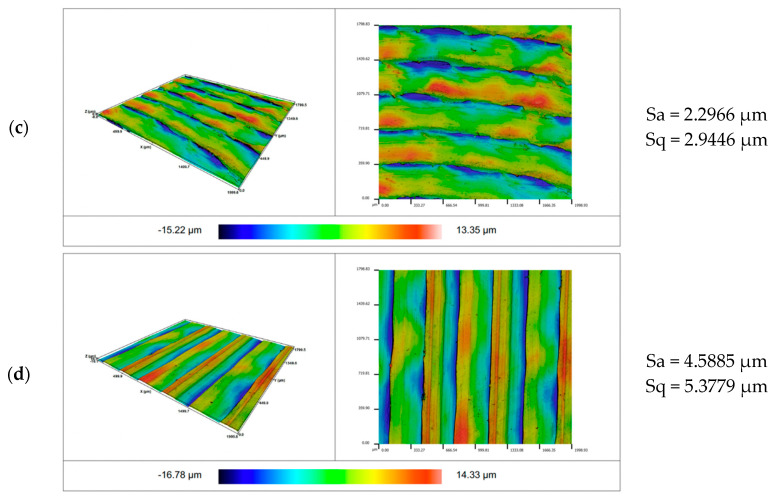
Isometric views of the surface roughness made with different configuration of patterns and material: (**a**) TPU circle; (**b**) TPU line; (**c**) PLA circle; and (**d**) PLA line.

Figure 11 shows the views of the exemplary surfaces obtained as a result of using different configuration of patterns and material. As a result of the conducted research, the influence of the analyzed input parameters on the surfaces roughness was observed. It was found that the Sa parameter for the linear pattern is greater than for the circular pattern. Comparing the effect of the Sa parameter on the material, it is greater for PLA filament. A similar trend in the surface roughness measurement results was observed for the Sq parameter, which was characterized by a strong correlation with the Sa parameter.

### 3.3. Roughness and Adhesion Summarised

**Figure 12:**
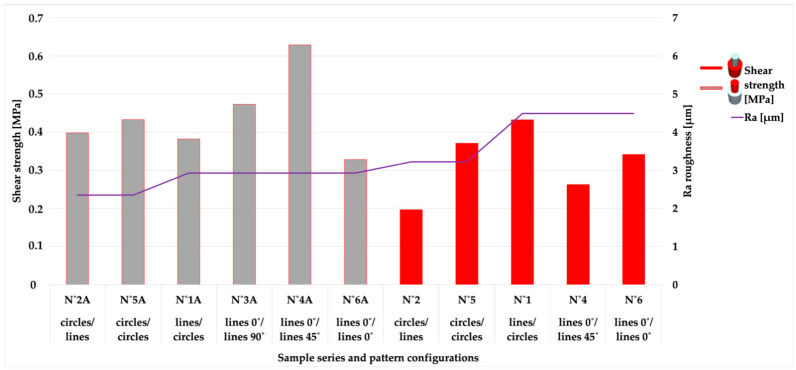
The relationship between roughness (Ra) and shear strength for each pattern and material combinations.

There were four possible patterns on the lower cylinders. Two patterns were overprinted on the TPU and PLA samples with a *Concentric* pattern (*Lines* and *Concentric*) while on samples with a *Lines* pattern. Four options of the pattern were printed (*Concentric* and *Lines* with different angles). The diagram (Figure 12) shows the relationship between roughness (R_a_) and shear strength. The arithmetic mean of the obtained roughness values was calculated for each sample. The results are summarized in ascending order concerning the values of the shear strength.

## 4. Discussion

Multi-material printing allows different filaments to be used to print parts in a single-step process. They are analyzing leads to the possibility of creating multifunctional parts with specific properties. However, printing from multiple materials will inevitably lead to the creation of boundary interfaces between different materials.

To broaden the understanding of boundary surfaces, this article describes a study that explored the effect of roughness on the adhesion strength between pairs of polymers. The following aspects were examined: pattern used for the top/bottom layer and printing the materials. Two sets of materials were tested (PLA-TPU and TPU-PLA) with six different patterns configurations for each. The results demonstrate that the printing order and surface pattern, due to the roughness of the material contact surface, influences the adhesion strength.

The bar chart (Figure 7) illustrates the maximum force in N for all sample series and therefore shows that the most significant force was applied to sample N°1 (PLA/TPU with Lines/ Concentric pattern). Moreover, the minor force was applied to the sample with the same material configuration but opposite patterns: N°2 (PLA/TPU with Concentric/ Lines pattern). The diagram shows (Figure 7) that the values achieved for the TPU/ PLA sample series, regardless of the pattern, are similar, unlike samples where printing order was reversed (PLA as bottom cylinder, and TPU as top). The results confirm this: the TPU/PLA sample is 4.75 when for PLA/TPU is 12.51. It can be concluded that printing on TPU is more predictable than printing on PLA, irrespective of the patterns.

Shear strength, shown in Figure 8, presents the values for each pair of samples with different patterns combinations on the contact surfaces. By analyzing pairs of samples (the same patterns, different order of materials), the results for the TPU/PLA samples were better or nearing the results for the PLA/TPU samples. Arithmetic means corroborates it: for TPU/PLA- 0.39 MPa, while for PLA/TPU- 0.37 MPa.

Subsequently, displacement at maximum force was presented. Among all pairs of samples, the TPU/PLA samples showed significantly higher values (average 3.4 mm) than the PLA/TPU (average 1.49 mm). Due to the hardness of the materials (in Shore D: TPU- 60, PLA- 83), the results of the shear tests proved the relationship between plasticity and adhesive strength. A knife was placed on the upper PLA cylinder during the tests (on the TPU/PLA samples). When force was applied, the TPU material underwent plastic deformation, which contributed to higher values of displacements.

The roughness of four sample variants was tested. There were four possible patterns on the lower cylinders PLA and TPU with a Concentric and Lines pattern. The thermoplastic polyurethane samples had a lower surface roughness (Figure 10). As shown in Figure 12, roughness has no direct effect on the adhesive strength. The best variants of patterns combinations were distinguished:

- No5A: TPU with a Concentric pattern—PLA with Concentric pattern
- No4A: TPU with a Lines 0° pattern—PLA with Lines 45° pattern
- No5: PLA with a Concentric pattern—TPU with Concentric pattern
- No1: PLA with a Line pattern—TPU with Concentric pattern

In the literature, the possibilities of combining different filaments in the FDM method have been investigated.

Lopes et al. [29] have proven the influence of TPU plasticity on the connection with PLA, which was confirmed in this study, especially considering the results for deformation. The TPU-PLA samples combine a flexible and rigid material. Thus, the part presents a flexible behavior provided by the TPU section. Tamburrino et al. [35] observed impediments while printing parts with flexible material at the bottom, providing an unstable base while printing. Scientists concluded that adhesion strength decreases if TPU is the first material to be printed. The statement is not confirmed with these studies; the results were presumably influenced by the sample size, printing parameters, and the selection of surface pattern. This leads to the conclusion that studies on multi-material printing are needed to enable designers, based on the literature, to select the most appropriate printing parameters for parts of a specific size and geometry.

## 5. Conclusions

The research revealed the best combination of surface development during 3D printing from two thermoplastic polymers. This combination has achieved the best strength in destructive shear tests. Due to the strong dependence between adhesion, printing parameters, size, and the geometry of samples, research about the combinations of PLA thermoplastic polymers with TPU in 3D printing is planned. Future research will focus on printing parameters influencing the quality of the connection, process temperatures, and preservation of material connections in samples with other geometries, considering strength, tensile, and bending tests of samples.

The significant advantages of FDM 3D printing are the options of printing fast and with a geometrical latitude. 3D printing is evolving, and research on it creates the possibility of faster, one-step production from many materials simultaneously. Multi-material printing requires well-conceived model construction, predicting the quality of materials connections and ways to improve it. Such research has broadened the knowledge of different filaments bonding and facilitated the work of model designs. Well-chosen printing parameters can prevent a drop in parts efficiency compared to component materials, which ultimately depend on the materials’ combination.

## Figures and Tables

**Table 1:** Mechanical properties of PLA and TPU, referring to the technical data sheet [44,45].

Mechanical Properties	Material	Typical Value	Test Method
Hardness	PLA	83 (Shore D)	Durometer
Tensile modulus	PLA	2.346 MPa	ISO 527 (1 mm/min)
Elongation at break	PLA	5.2%	ISO 527 (50 mm/min)
Hardness	TPU	60 (Shore D)	ISO 7619–1
Tensile strength	TPU	53.7 MPa	DIN 53504 (200 mm/min)
Elongation at break	TPU	318%	DIN 53504 (200 mm/min)

**Table 2:** Printing parameters.

Print Setting	Extruder 1: PLA	Extruder 2: TPU
Layer Height	0.2 mm	0.2 mm
Top/Bottom Line Width	0.35 mm	0.35 mm
Wall Line Court	3	4
Top/Bottom Layers	5	6
Infill Density	70%	100%
Infill Pattern	Triangles	Grid
Printing Temperature	200 °C	210 °C
Print Speed	70 mm/s	30 mm/s

**Table 3:** 3D model of the printed parts with force point variants, pattern and material configurations, and the number of printed samples.

A 3D Model with A Visible Pattern	Pattern Configuration	Material Configuration/N°	
		Bottom: PLA Top: TPU	Bottom: TPU Top: PLA
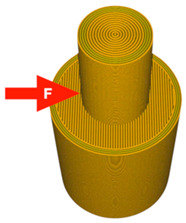	Bottom part: Lines Top part: Concentric	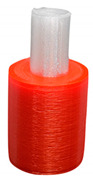 N°−1	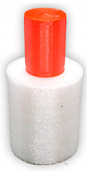 N°−1A
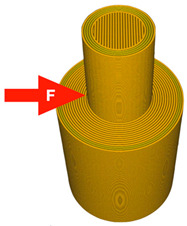	Bottom part: Concentric Top part: Lines	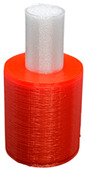 N°−2	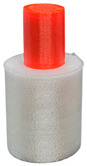 N°−2A
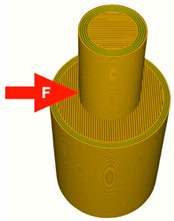	Bottom part: Lines (Line direction: 0°) Top part: Lines (Line direction: 90°)	Failure N°−3	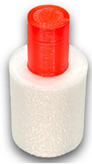 N°−3A
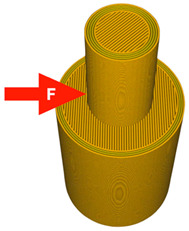	Bottom part: Lines (Line direction: 0°) Top part: Lines (Line direction: 45°)	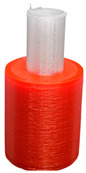 N°−4	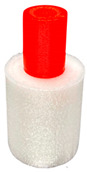 N°−4A
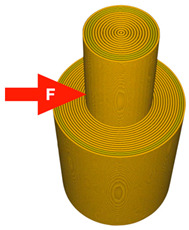	Bottom part: Concentric Top part: Concentric	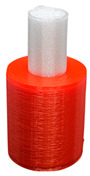 N°−5	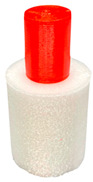 N°−5A
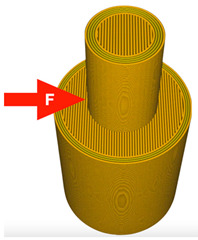	Bottom part: Lines 0° Top part: Lines 0°	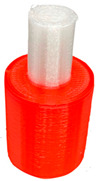 N°−6	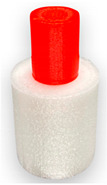 N°−6A

## Data Availability

Not applicable.

## References

[1] Liu Y., Chen J., Shang E., Chen Y. (2020). Process based modeling of energy consumption for multi-material FDM 3D printing. Chin. J. Mech. Eng..

[2] Yadav D., Chhabra D., Garg R.K., Ahlawat A., Phogat A. (2020). Optimization of FDM 3D printing process parameters for multi-material using artificial neural network. Mater. Today Proc..

[3] Anitha R., Arunachalam S., Radhakrishnan P. (2001). Critical parameters influencing the quality of prototypes in fused deposition modelling. J. Mater. Process. Technol..

[4] Garzon-Hernandez S., Garcia-Gonzalez D., Jérusalem A., Arias A. (2020). Design of FDM 3D printed polymers: An experimental-modelling methodology for the prediction of mechanical properties. Mater. Des..

[5] Citarella R., Giannella V. (2021). Additive Manufacturing in Industry. Appl. Sci..

[6] Budzik G., Woźniak J., Paszkiewicz A., Przeszłowski Ł., Dziubek T., Dębski M. (2021). Methodology for the Quality Control Process of Additive Manufacturing Products Made of Polymer Materials. Materials.

[7] Kusoglu I., Doñate-Buendía C., Barcikowski S., Gökce B. (2021). Laser Powder Bed Fusion of Polymers: Quantitative Research Direction Indices. Materials.

[8] Popescu D., Zapciu A., Amza C., Baciu F., Marinescu R. (2018). FDM process parameters influence over the mechanical properties of polymer specimens: A review. Polym. Test..

[9] Haghsefat K., Eng M., Tingting L. FDM 3D Printing Technology and Its Fundemental Properties. Proceedings of the International Conference on Innovation and Research in Engineering Sciences.

[10] Bergonzi L., Vettori M., Stefanini L., D’Alcamo L. (2021). Different infill geometry influence on mechanical properties of FDM produced PLA. IOP Conference Series: Materials Science and Engineering, Proceedings of the 49th AIAS Conference (AIAS 2020), Genova, Italy, 2–5 September 2020.

[11] Buj-Corral I., Bagheri A., Domínguez-Fernández A., Casado-López R. (2019). Influence of infill and nozzle diameter on porosity of FDM printed parts with rectilinear grid pattern. Procedia Manuf..

[12] Yahamed A., Ikonomov P., Fleming P.D., Pekarovicova A., Gustafson P., Alden A.Q., Alrafeek S. (2016). Mechanical properties of 3D printed polymers. J. Print Media Technol. Res..

[13] Taczała J., Czepułkowska W., Konieczny B., Sokołowski J., Kozakiewicz M., Szymor P. (2020). Comparison of 3D printing MJP and FDM technology in dentistry. Arch. Mater. Sci. Eng..

[14] Lüchtenborg J., Burkhardt F., Nold J., Rothlauf S., Wesemann C., Pieralli S., Wemken G., Witkowski S., Spies B. (2021). Implementation of Fused Filament Fabrication in Dentistry. Appl. Sci..

[15] Jeon B., Han J.W., Lee K.S., Cha S.W. (2012). Improvement of the Mechanical Properties of Biodegradable Polymers Using a Microcellular Foaming Process and Natural By-Products. Polym. Technol. Eng..

[16] Tao Y., Shao J., Li P., Shi S.Q. (2019). Application of a thermoplastic polyurethane/polylactic acid composite filament for 3D-printed personalized orthosis. Mater. Teh..

[17] Mamiński M., Novák I., Mičušík M., Małolepszy A., Toczyłowska-Mamińska R. (2021). Discharge Plasma Treatment as an Efficient Tool for Improved Poly(lactide) Adhesive–Wood Interactions. Materials.

[18] Lee Y.-H., Kang B.-K., Kim H.-D., Yoo H.-J., Kim J.-S., Huh J.-H., Jung Y.-J., Lee D.-J. (2009). Effect of hot pressing/melt mixing on the properties of thermoplastic polyurethane. Macromol. Res..

[19] Tabuani D., Bellucci F., Terenzi A., Camino G. (2012). Flame retarded Thermoplastic Polyurethane (TPU) for cable jacketing application. Polym. Degrad. Stab..

[20] Mrówka M., Szymiczek M., Machoczek T., Pawlyta M. (2021). Influence of the Halloysite Nanotube (HNT) Addition on Selected Mechanical and Biological Properties of Thermoplastic Polyurethane. Materials.

[21] He X., Zhou J., Jin L., Long X., Wu H., Xu L., Gong Y., Zhou W. (2020). Improved Dielectric Properties of Thermoplastic Polyurethane Elastomer Filled with Core–Shell Structured PDA@TiC Particles. Materials.

[22] Lee H., Eom R.-I., Lee Y. (2019). Evaluation of the Mechanical Properties of Porous Thermoplastic Polyurethane Obtained by 3D Printing for Protective Gear. Adv. Mater. Sci. Eng..

[23] Sambruno A., Bañon F., Salguero J., Simonet B., Batista M. (2019). Kerf Taper Defect Minimization Based on Abrasive Waterjet Machining of Low Thickness Thermoplastic Carbon Fiber Composites C/TPU. Materials.

[24] Ji X., Gao F., Geng Z., Li D. (2021). Fabrication of thermoplastic polyurethane/polylactide shape-memory blends with tunable optical and mechanical properties via a bilayer structure design. Polym. Test..

[25] Lis-Bartos A., Smieszek A., Frańczyk K., Marycz K. (2018). Fabrication, Characterization, and Cytotoxicity of Thermoplastic Polyurethane/Poly(lactic acid) Material Using Human Adipose Derived Mesenchymal Stromal Stem Cells (hASCs). Polymers.

[26] Hu Q., Suihong L., Yan G., Zhicheng S. (2021). Topological Structure Design and Fabrication of Biocompatible PLA/TPU/ADM Mesh with Appropriate Elasticity for Hernia Repair. Macromol. Biosci..

[27] Liu X., Zhou L., Heng P., Xiao J., Lv J., Zhang Q., Hickey M.E., Tu Q., Wang J. (2018). Lecithin doped electrospun poly(lactic acid)-thermoplastic polyurethane fibers for hepatocyte viability improvement. Colloids Surf. B Biointerfaces.

[28] Han Y., Kim J. (2018). A Study on the Mechanical Properties of Knit Fabric Using 3D Printing—Focused on PLA, TPU Filament. J. Fash. Bus..

[29] Lopes L., Silva A., Carneiro O. (2018). Multi-material 3D printing: The relevance of materials affinity on the boundary interface performance. Addit. Manuf..

[30] Schwartz J.J., Boydston A.J. (2019). Multimaterial actinic spatial control 3D and 4D printing. Nat. Commun..

[31] Mohammed M.I., Tatineni J., Cadd B., Peart G., Gibson I. Advanced auricular prosthesis development by 3D modelling and multi-material printing. Proceedings of the International Conference on Design and Technology (DesTech 2016).

[32] Yin J., Li M., Dai G., Zhou H., Ma L., Zheng Y. (2021). 3D Printed Multi-material Medical Phantoms for Needle-tissue Interaction Modelling of Heterogeneous Structures. J. Bionic Eng..

[33] Skylar-Scott M.A., Mueller J., Visser C.W., Lewis J.A. (2019). Voxelated soft matter via multimaterial multinozzle 3D printing. Nature.

[34] Ge Q., Sakhaei A.H., Lee H., Dunn C.K., Fang N.X., Dunn M.L. (2016). Multimaterial 4D Printing with Tailorable Shape Memory Polymers. Sci. Rep..

[35] Tamburrino F., Graziosi S., Bordegoni M. (2019). The influence of slicing parameters on the multi-material adhesion mechanisms of FDM printed parts: An exploratory study. Virtual Phys. Prototyp..

[36] Rahmati S., Vahabli E. (2015). Evaluation of analytical modeling for improvement of surface roughness of FDM test part using measurement results. Int. J. Adv. Manuf. Technol..

[37] Garg A., Bhattacharya A., Batish A. (2016). On Surface Finish and Dimensional Accuracy of FDM Parts after Cold Vapor Treatment. Mater. Manuf. Process..

[38] Ahn D., Kweon J.-H., Kwon S., Song J., Lee S. (2009). Representation of surface roughness in fused deposition modeling. J. Mater. Process. Technol..

[39] Bouzouita A., Notta-Cuvier D., Raquez J.-M., Lauro F., Dubois P., Di Lorenzo M., Androsch R. (2017). Poly(lactic acid)-Based Materials for Automotive Applications. Industrial Applications of Poly(lactic acid). Advances in Polymer Science.

[40] Bouzouita A. (2016). Elaboration of Polylactide-Based Materials for Automotive Application: Study of Structure-Process-Properties Interactions Amani Bouzouita. Ph.D. Thesis.

[41] Fort T., Bruns K., Bichel A., Miller J., Vanstrom J.R., Koziel J.A. (2018). Puncture and Cut Resistant Glove Testing.

[42] (2017). Higher Dimension Materials, Inc. Cut, Abrasion and/or Puncture Resistant Knitted Gloves. U.S. Patent.

[43] Chari S., Haines T., Varghese P., Economidis A. (2009). Are non-slip socks really ’non-slip’? An analysis of slip resistance. BMC Geriatr..

[44] Filamentum, Flexfill TPU 98A, TDS. http://www.fillamentumautomotive.com/wp-content/uploads/2020/10/Technical-Data-Sheet_Flexfill-TPU-98A_26082019.pdf.

[45] Ultimaker PLA, TDS. https://support.ultimaker.com/hc/en-us/articles/360011962720.

[46] Taczała J., Rak K., Sawicki J., Krasowski M. (2021). Numerical Analysis of the Bond Strength between Two Methacrylic Polymers by Surface Modification. Materials.

[47] Surface Texture from Ra to Rz. https://www.mmsonline.com/columns/surface-texture-from-ra-to-rz.

